# Altered Gut Microbiome and Environmental Factors Associated with Development of Eczema in Hong Kong Infants: A 4-Month Pilot Study

**DOI:** 10.3390/ijerph17207634

**Published:** 2020-10-20

**Authors:** Carmen Wing Han Chan, Judy Yuet Wa Chan, Ting Fan Leung, Kai Chow Choi, Stephen Kwok Wing Tsui, Cho Lee Wong, Ka Ming Chow

**Affiliations:** 1 The Nethersole School of Nursing, The Chinese University of Hong Kong, Shatin, New Territories, Hong Kong; whchan@cuhk.edu.hk (C.W.H.C.); kchoi@cuhk.edu.hk (K.C.C.); jojowong@cuhk.edu.hk (C.L.W.); kmchow@cuhk.edu.hk (K.M.C.); 2 Department of Paediatrics, The Chinese University of Hong Kong, Shatin, New Territories, Hong Kong; tfleung@cuhk.edu.hk; 3 Hong Kong Hub of Paediatric Excellence, The Chinese University of Hong Kong, Shatin, New Territories, Hong Kong; 4 School of Biomedical Sciences, The Chinese University of Hong Kong, Shatin, New Territories, Hong Kong; kwtsui@cuhk.edu.hk

**Keywords:** eczema, gut microbiome, maternal diet, environmental factors

## Abstract

Eczema is a multifactorial skin disease that affects 20% of children worldwide and has a complex relationship with microbial, nutritional, parental and environmental factors. In this study, we investigated the potential association of eczema with the gut microbiome and environmental factors. One hundred and fifty-two newborn subjects and their mothers were recruited within 10 days postnatally at the Prince of Wales Hospital in Hong Kong, China and asked to complete questionnaires on allergies, maternal diet and environmental assessment at enrolment. Then, the participants were classified as with or without eczema at four months after birth based on the Comprehensive Early Childhood Allergy Questionnaire (CECAQ) and SCORing Atopic Dermatitis (SCORAD) index (*n* = 48, with 24 in each group). Stool samples were collected from both groups at the same time. Microbial DNA was extracted from each stool sample, and 16S rRNA sequencing was performed to analyze the gut microbiome profiles of the subjects. Our results indicated that the abundance of *Bifidobacterium* was significantly higher in the eczema group than in the control group (*p* = 0.04). A multivariable logistic regression analysis was conducted, and the results showed that the father’s education level and maternal intake of cereal products and nutritional supplements during pregnancy were associated with the development of eczema (*p* = 0.008, 0.032 and 0.015, respectively). In conclusion, this study provided preliminary information about the potential risk factors of eczema development in Hong Kong infants in support of a future full study.

## 1. Introduction

Eczema, a chronic and relapsing type of skin inflammation, affects approximately 20% of children (mainly infants under one year of age) and 3% of adults worldwide [1,2]. Most cases (~70%) start in early childhood between zero and five years of age [3], with the symptoms developing within the first two years. The number of eczema cases in Hong Kong is increasing by 0.12% per year [4]. The dermatitis associated with asthma requires diligent treatment regiments, which impose financial and mental burdens on the sufferers’ families. Up to 80% of patients with eczema will develop asthma or allergic rhinitis in their lifetime [2]. For this reason, it is important to identify the factors affecting eczema development in early childhood, which may be significant at the beginning of the “atopic march”.

The etiology of eczema is multifaceted and not yet completely characterized. The environmental factors vary among countries, resulting in a variable prevalence in different locations. Another crucial factor in eczema is the patients’ microbiomes, which play an important role in the development of the immune system [5]. A previous study on *Lactobacillus rhamnosus* demonstrated a beneficial effect of this species in reducing the risk and severity of eczema. One explanation involves the recognition of Toll-like receptors (TLRs) expressed on T cells [6]. A recent integrative review reported that the infant gestational age, delivery mode and feeding type affected the gut microbiome composition [7]. Significant differences in skin microbiomes were also identified between children with eczema and adults with eczema [8]. However, in Chinese populations, particularly in Hong Kong, China, the association between the gut microbiota and the development of eczema is yet to be defined.

The development of eczema may also depend on the daily environment, socio-financial factors and maternal factors. It was reported that exposure to certain antigens appears to increase the tolerance and lower the risk of eczema in newborn children [9]. Domestic conditions such as pet ownership, the presence of siblings and the use of probiotics and supplements may also influence the occurrence of allergies [10]. Socioeconomic components, including the parental educational and stress levels, may conceivably be associated with the disease [11,12]. Further investigations are required to confirm the linkage of all of these factors with eczema development in infants.

Considering the possible interplay of the gut microbiota and environmental influences on the development of eczema, and the lack of evidence from Hong Kong sources in this research area, this study investigated the potential associations of eczema with the gut microbiome and environmental factors in a Hong Kong population.

## 2. Materials and Methods

### 2.1. Subjects

We recruited 152 newborn infants consecutively from January to December 2017 in the Department of Obstetrics and Gynaecology of the Prince of Wales Hospital in Hong Kong, China, a regional acute government hospital serving the Eastern New Territories of Hong Kong. Written consent forms for study participation were signed by the parents. We excluded subjects who did not regularly live in Hong Kong who were admitted to the neonatal intensive care unit; had gastrointestinal disorders after birth and whose mothers had fever, infection or were taking antibiotics. At 4 months after birth, the recruited infants were assessed for allergic conditions. Twenty-four infants reported to have eczema, and 24 infants without eczema were selected randomly and included in the analysis. This study was approved by the Joint Chinese University of Hong Kong–New Territories East Cluster Clinical Research Ethics Committee (2016.321).

### 2.2. Study Design

**Figure 1:**
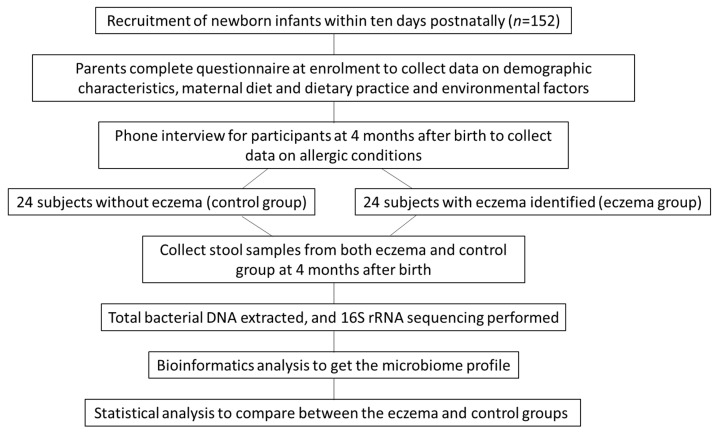
Diagram showing the procedural flow of the study.

Figure 1 shows the procedural flow of this study. Newborn infants were recruited into a birth cohort study. Data on demographic variables, the maternal diet during pregnancy, the maternal use of supplements during pregnancy and environmental factors were collected at enrolment. At 4 months after birth, the allergic conditions of the participants were assessed using a modified parent proxy version of the Comprehensive Early Childhood Allergy Questionnaire (CECAQ) developed by Minasyan and colleagues [13]. The severity of the allergic conditions was evaluated by physicians using the SCORing Atopic Dermatitis (SCORAD) index, which was designed to identify cases of eczema [14]. At the same time, fecal samples were collected from the participants for a microbiome analysis. Finally, 24 participants confirmed to have eczema and 24 without eczema were randomly selected for analysis (*n* = 48, with 24 in each group).

### 2.3. Questionnaire Data

The questionnaire completed at enrollment consisted of three main parts: demographic data, maternal diet and environmental assessment.

#### 2.3.1. Demographic Data

Sociodemographic and physical characteristics, including the infant’s gender, birth weight, gestational age, mode of delivery, parents’ level of education and other data, were self-reported at enrolment. All were categorical variables, except for the maternal age.

#### 2.3.2. Maternal Diet

Maternal diet and supplement use during pregnancy were assessed at enrolment using a parent proxy dietary practice questionnaire adapted from the Chinese version of the eating-habit module of the Behavioural Risk Factor Surveillance System (BRFSS), Hong Kong Centre for Health Protection.

#### 2.3.3. Environmental Factors and Allergy Data

Data on allergic diseases, family history of allergy, parental socioeconomic status, parental smoking, air quality and infants’ feeding patterns were collected using a modified parent proxy version of the CECAQ [13] at enrolment. The questionnaire was further reviewed by an expert panel of pediatricians, geneticists, pediatric nurses, academics and medical scientists. The feasibility, appropriateness and acceptability of the questionnaire were confirmed by five proxy parents of eczematous children. An average of 7 min was required to complete the questionnaire.

#### 2.3.4. Gut Microbiome Analysis

Stool samples from the 48 participating infants were collected from the 4-month-old infants’ diapers using sterile cotton swabs, and each sample was suspended in 0.6 mL of lysis buffer supplied with the TIANamp stool DNA kit (Tiangen, Beijing, China). The stool samples were stored immediately in a freezer at −80 °C before DNA preparation. Total microbial DNA was extracted from the stool using the TIANamp stool DNA kit according to the manufacturer’s instructions. The quantity and quality of the extracted DNA were verified by a Bioanalyser 2100 (Agilent Technologies, Santa Clara, CA, USA). Metagenomic libraries of each sample were then constructed using PCR primers flanking the V3-V4 region of the 16S ribosomal gene. Following PCR with a high-fidelity enzyme, amplicons with different barcodes were analyzed using the Bioanalyser 2100 (Agilent Technologies, Santa Clara, CA, USA) and a Qubit spectrophotometer (Thermo Fisher Scientific, Waltham, MA, USA). Equimolar amounts of amplicon libraries were pooled for next-generation sequencing using the Illumina MiSeq platform (Thermo Fisher Scientific, Waltham, MA, USA).

The metagenomic data set was analyzed using an open-source bioinformatics tool. Briefly, cleaned sequence reads were trimmed and aligned to the bacterial subset SILVA alignment, followed by filtering to remove vertical gaps. The reads were then screened for potential chimeras using the uchime method and, finally, classified using the Ribosomal Database Project’s naïve Bayesian classifier (RDP-NBC) (GitHub, San Francisco, USA) training set for mothur. Differentially abundant bacterial taxa, principal component analysis (PCoA) loadings, species richness (Sobs), Shannon’s diversity (H’) and evenness (EH) and Simpson’s diversity (SD) were calculated using the mothur workflow.

### 2.4. Statistical Analysis

Categorical and continuous data were summarized and are presented as frequencies (percentage) and mean (standard deviation) respectively. Comparisons of the numbers of observed bacterial species in the stools from the control and eczema groups according to the Shannon diversity index were conducted using a *t*-test, whereas comparisons of the abundance of various bacterial genera in the control and eczema groups were performed using Mann-Whitney tests. Univariate analyses between the development of eczema in infants and the demographic, maternal diet and environmental factors were performed using the independent *t*, Mann-Whitney, chi-square and Fisher’s exact tests, as appropriate. Those variables with *p*-values < 0.1 in univariate analyses were selected as candidate independent variables for a backward multivariable logistic regression to delineate the factors independently associated with the presence of eczema. All statistical analyses were performed using SPSS version 24 (IBM Corp., Armonk, NY, USA), and all statistical tests involved were two-sided with the level of significance set at 0.05.

## 3. Results

### 3.1. General Characteristics and Occurrence of Eczema

Table 1 shows the demographic data of the 48 participants, which were collected from their parents at enrolment through self-reported questionnaires. The results indicate that no association was found between most of the individual characteristics (e.g., birth weight, mode of delivery or breastfeeding rate) and the occurrence of eczema. However, the father’s education was found to be significantly associated with the occurrence of eczema in infants (*p* = 0.009) when continuous and categorical variables were compared between the two groups using the independent *t*-test and Pearson chi-square test, respectively (Table 1).

### 3.2. Association of Gut Microbiome and Eczema

**Figure 2:**
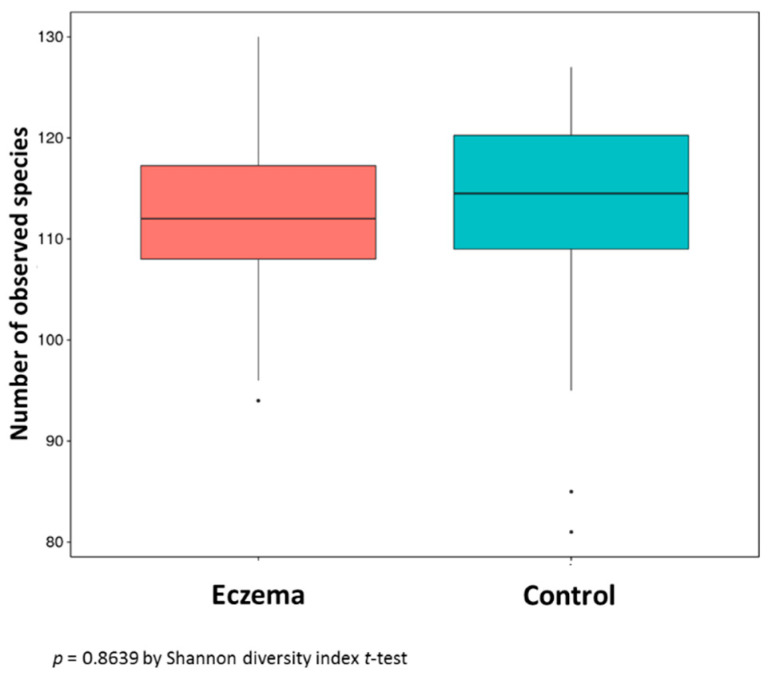
Number of observed bacterial species in the stools from 24 eczema cases and 24 control subjects.

Genomic DNA was prepared from the 48 stool samples, and 16S rRNA sequencing was performed to profile the microbiome diversity. The results showed that the numbers of bacterial species in the stool samples from the eczema group and control group were similar without a significant difference (*p* = 0.8639) (Figure 2). However, the abundance of *Bifidobacterium* (genus) in the eczema group (mean ± SD = 18.98% ± 15.03%) was significantly higher than that in the control group (mean ± SD = 12.49% ± 12.24) (* *p* = 0.04036) (Table 2).

### 3.3. Association of Maternal Diets, Use of Supplements, Environmental Factors and Eczema

The maternal diets and supplement uses during pregnancy were assessed at enrolment by the parent proxy dietary practice questionnaire, which was adapted from the Chinese version of the eating-habit module of the BRFSS. The data collected included the maternal consumption of various foods and supplements (e.g., fruit, vegetables and dairy products) and the mothers’ eating practices during pregnancy (Table 3 and Table 4). The results indicated that the consumption of dairy products and nutritional supplements during pregnancy were associated with the development of eczema (*p* = 0.007 and *p* = 0.037, respectively) when continuous and categorical variables were compared between the two groups using the independent *t*-test and Pearson chi-square test, respectively.

### 3.4. Association of Environmental Factors and Eczema

Environmental factors such as smoking at home, presence of pets at home, air quality and infection during pregnancy were assessed by the questionnaire. The results in Table 5 show that, in the univariate analysis, the presence of a pet at home during pregnancy was associated with the occurrence of eczema (*p* = 0.029).

### 3.5. Multivariate Analysis

Univariate analyses between the development of eczema in infants and their demographics, maternal diet and environmental factors were performed using independent *t*, Mann-Whitney, chi-square and Fisher’s exact tests, as appropriate. Subsequently, those variables with *p*-values < 0.1 in the univariate analyses were selected as candidate independent variables for a backwards multivariable logistic regression to delineate the factors independently associated with the presence of eczema. After a statistical analysis of all outcome variables simultaneously, the results showed that the father’s education level and maternal intake of cereal products and nutritional supplements during pregnancy were associated with the development of eczema (*p* = 0.008, 0.032 and 0.015, respectively) (Table 6).

## 4. Discussion

Eczema is the most common chronic inflammatory skin disorder in children [15,16]. Its prevalence has increased in recent decades and imposed both socio-burden and economic burdens on healthcare systems [17,18,19]. Thus, a better knowledge of the risk factors for childhood eczema would be useful in public health [20]. This study identified some potential risk factors for eczema, including the gut microbiome profiles and environmental factors, in the early lives of infants in Hong Kong.

Eczema is multifactorial and caused by a variety of factors, including genetic, parental and environmental influences. Among them, the gut microbial environment at infancy is important in terms of its role in childhood immune programming. In this study, the frequency and diversity of the microbial distributions were examined in infants at four months of age. Total microbial DNA was extracted from the stools of 24 subjects each in a control group and an eczema group. Then, 16S rRNA sequencing was performed, and the data were analyzed using bioinformatics tools. The results showed no significant difference in the number of bacterial species between the eczema group and control group (*p* = 0.8639) (Figure 2). In contrast, an earlier study reported a difference in the fecal microbial community diversity between healthy infants and those with eczema at very early stages (one to four months of age). Reduced microbial diversity is associated with the development of eczema in early life [21]. One possible reason for the discrepancy between the two studies is the infants’ diets. Any difference in the breastfeeding rates and maternal diets between different populations may influence the infants’ gut microbiome compositions. Other possible reasons for this difference may include dietary practices and differences in the climate and postnatal practices. In this study, no association was found between eczema development and breastfeeding or the delivery mode (*p* > 0.05). However, the impact of these factors on the microbiome should be investigated in future studies.

Nonetheless, this study did find a difference between the compositions of the fecal microbiotic communities of the control group and eczema group. When individual genera were analyzed, the abundance of *Bifidobacterium* in the eczema group was significantly higher than that in the control group (*p* < 0.05). This result was in contrast to the findings from a study of Swedish infants by Abrahamsson et al., wherein *Bifidobacterium* was more abundant in healthy infants than in those with eczema [22]. This difference may be due to interethnic variations. In another study in Hong Kong, the gut microbiome composition of infants at four weeks was analyzed [23]. In that study, no significant differences in the most abundant genera (≥1%), including *Bacteroides*, *Escherichia*, *Klebsiella, Bifidobacterium*, *Streptococcus* and *Lactobacillus*, were detected between infants with eczema and healthy controls. Among the less-abundant genera (relative abundance <1%), *Campylobacter* was more abundant in infants with eczema (median 0.008%, interquartile range (IQR) 0.003–0.022%)) than in controls (median 0.001%, IQR 0.001–0.004%), while *Roseburia* was less abundant in participants with eczema (median 0%, IQR 0–0.063%) than in controls (median 0.055%, IQR 0.002–0.270%). However, the number of subjects in that study was only 25; additional larger-scale studies of Chinese populations are required to yield more definitive results and explain the differences between studies regarding the association between the gut microbiome and infant eczema.

The development of eczema is affected by the skin barrier function and the immune system, which, in turn, are sensitive to environmental factors, such as feeding patterns, maternal diet and supplement use during pregnancy, air quality and others. Any discordance between these factors and the early developmental requirements of the infant’s immune system may contribute to the development of allergic diseases [24]. In this study, the associations of maternal and environmental factors with the development of eczema were investigated. When the univariate odds ratio was adjusted for other significant factors obtained from the backwards logistic regression analysis, three factors were concluded to be associated with the occurrence of eczema (Table 6): the education level of the father, amount of cereal foods consumed daily by the mother during pregnancy and nutritional supplements taken by the mother during pregnancy. Among these three factors, the relationship between the education level of the father and the development of eczema would seem to be indirect. Infants whose fathers had a higher-level education were found to have a higher chance of developing eczema (OR_A_ (odds ratio adjusted) (95% CI) = 9.93 (1.83–53.71), *p* = 0.008). The father’s educational background may directly affect the infant’s living environment (e.g., the level of hygiene). This may be important in the context of the hygiene hypothesis proposed by Strachan [25], which suggests that a lower incidence of infections transmitted through contact with unhygienic environments in early childhood could be a cause of the rise in allergic diseases. The other two risk factors, namely the maternal consumption of cereal products and nutritional supplements during pregnancy, have more apparent direct relationships with infants’ health. Early sensitization to food allergens occurs through breast milk, skin contact and/or inhalation and may explain why some infants show an allergic response to specific proteins despite having never ingested them [26]. Our results indicated that infants had a greater chance of developing eczema when their mothers took supplements (OR_A_(95% CI) = 10.75 (1.57–73.46), *p* = 0.015) or ate smaller amounts of cereal products (OR_A_(95% CI) = 0.10 (0.01–0.82), *p* = 0.032). Although most studies investigating the underlying immunomodulatory mechanisms have focused on postnatal microbial exposure [27,28,29], accumulating evidence shows that the maternal microbial environment during pregnancy is also important to childhood immune programming [30,31,32]. During pregnancy, the maternal microbiome strongly affects the child’s immune development because of the interplay between the immune system of the mother and that of her offspring [33]. In our study, the nutritional supplements taken by the mothers during pregnancy included formulated powders with minerals and vitamins, algae oil pills and fish oil pills. It remains unknown how these supplements affected the maternal microbiome. A more profound understanding of these communicative processes between the maternal and offspring microbiomes and their implications for immunity would conceivably allow us to identify adequate preventive measures to combat the allergy epidemic. Therefore, it appears to be likely that particular components during early life can contribute to normal immune development via multiple direct and indirect pathways, thereby increasing or reducing the risk of allergic manifestations.

This study investigates the influences of the microbiome and environmental factors on the development of eczema in infants at four months after birth. A potential limitation of this study is the short duration. In future, a full study lasting two years with several interim time points will be conducted to examine the situation over a longer period.

## 5. Conclusions

This study provided preliminary data on the factors affecting the development of eczema. The parental education level, maternal consumption of cereal products and maternal intake of nutritional supplements during pregnancy were associated with eczema. Further studies with greater numbers of subjects are required to consolidate these results.

## Figures and Tables

**Table 1:** Demographic characteristics of the participants (*n* = 48).

		Eczema		
Demographic Characteristics	All (*n* = 48)	No (*n* = 24)	Yes (*n* = 24)	*p*-Value
Mother’s age (years) ^†^	31.9 (3.9)	31.5 (4.5)	32.4 (3.5)	0.394 ^#^
Sex of the infant				
Female	25 (52.1%)	15 (62.5%)	10 (41.7%)	0.149 ^#^
Male	23 (47.9%)	9 (37.5%)	14 (58.3%)	
Low birth weight (<2.5 kg)				
No	46 (95.8%)	24 (100.0%)	22 (91.7%)	0.489 ^ψ^
Yes	2 (4.2%)	0 (0.0%)	2 (8.3%)	
Gestational age (weeks)				
37–38	13 (27.1%)	8 (33.3%)	5 (20.8%)	0.494 ^ψ^
39–40	29 (60.4%)	14 (58.3%)	15 (62.5%)	
>40	6 (12.5%)	2 (8.3%)	4 (16.7%)	
Mode of delivery				
Vaginal	38 (79.2%)	19 (79.2%)	19 (79.2%)	0.999 ^#^
Cesarian section	10 (20.8%)	5 (20.8%)	5 (20.8%)	
The infant is the first child				
No	25 (52.1%)	14 (58.3%)	11 (45.8%)	0.386 ^#^
Yes	23 (47.9%)	10 (41.7%)	13 (54.2%)	
Number of siblings of the infant				
0	24 (50.0%)	11 (45.8%)	13 (54.2%)	0.882 ^ψ^
1	22 (45.8%)	12 (50.0%)	10 (41.7%)	
2	2 (4.2%)	1 (4.2%)	1 (4.2%)	
Mother’s education				
Secondary	25 (52.1%)	15 (62.5%)	10 (41.7%)	0.149 ^#^
Tertiary	23 (47.9%)	9 (37.5%)	14 (58.3%)	
Father’s education				
Secondary	21 (43.8%)	15 (62.5%)	6 (25.0%)	0.009 ^#^
Tertiary	27 (56.3%)	9 (37.5%)	18 (75.0%)	
Monthly household income (HK$)				
10,000–19,999	7 (14.6%)	4 (16.7%)	3 (12.5%)	0.233 ^ψ^
20,000–29,999	11 (22.9%)	7 (29.2%)	4 (16.7%)	
30,000–59,999	23 (47.9%)	12 (50.0%)	11 (45.8%)	
≥60,000	7 (14.6%)	1 (4.2%)	6 (25.0%)	

Data marked with ^†^ are presented as mean (standard deviation); all others are presented as frequency (%). ^#^ Continuous and categorical variables were compared between the two groups using the independent *t*-test and Pearson chi-square test, respectively. Those marked with ^ψ^ were compared using Fisher’s exact test.

**Table 2:** The relative abundances of the dominant 20 bacterial genera in stool samples obtained at four months of age from 24 eczema cases and 24 control subjects.

	Control		Eczema		
Genus	Mean ± S.D.	Median (IQR)	Mean ± S.D.	Median (IQR)	*p*-Value ^#^
*Klebsiella*	3.33 ± 4.63	1.62 (0.65–3.59)	6.69 ± 13.47	2.90 (1.21–5.76)	0.219
*Escherichia*	27.12 ± 15.66	25.26 (13.85–39.77)	13.97 ± 17.30	17.36 (12.53–31.45)	0.418
*Lactobacillus*	3.45 ± 11.10	0.47 (0.08–1.25)	1.70 ± 2.34	0.62 (0.16–2.06)	0.503
*Bifidobacterium*	12.49 ± 12.24	6.41 (4.61–14.58)	18.98 ± 15.03	13.21 (7.90–23.07)	0.040 *
*Bacteroides*	15.10 ± 8.83	14.62 (9.22–19.15)	13.00 ± 11.43	9.97 (7.72–13.88)	0.097
*Clostridium*	6.66 ± 9.75	3.16 (0.99–8.33)	3.42 ± 6.42	1.26 (0.38–3.38)	0.112
*Parabacteroides*	5.73 ± 8.92	2.62 (1.61–5.91)	5.17 ± 6.57	3.32 (1.19–7.05)	0.772
*Collinsella*	2.06 ± 5.37	0.36 (0.17–1.07)	2.04 ± 3.90	0.61 (0.25–1.99)	0.144
*Ruminococcaceae*	0.38 ± 0.57	0.19 (0.02–0.56)	1.18 ± 4.81	0.09 (0.03–0.40)	0.465
*Streptococcus*	3.17 ± 4.76	1.85 (0.91–3.03)	2.78 ± 3.34	2.02 (0.92–3.77)	0.928
*Ruminococcus*	0.66 ± 0.70	0.40 (0.23–0.88)	1.71 ± 4.00	0.55 (0.21–1.14)	0.575
*Veillonella*	2.64 ± 3.25	1.11 (0.58–4.05)	3.42 ± 4.31	1.42 (0.72–6.32)	0.430
*Megamonas*	0.78 ± 3.27	0.02 (0.00–0.20)	0.09 ± 0.16	0.02 (0.01–0.12)	0.728
*Akkermansia*	1.78 ± 4.14	0.09 (0.01–0.63)	0.08 ± 0.10	0.03 (0.01–0.16)	0.089
*Hungatella*	1.30 ± 2.69	0.27 (0.12–1.15)	0.73 ± 1.07	0.18 (0.05–1.23)	0.358
*Lachnoclostridium*	1.03 ± 1.87	0.38 (0.11–0.94)	0.95 ± 1.87	0.35 (0.09–0.92)	0.646
*Megasphaera*	0.40 ± 1.53	0.04 (0.02–0.14)	0.45 ± 1.21	0.07 (0.03–0.13)	0.258
*Enterococcus*	1.17 ± 1.49	0.68 (0.31–1.37)	1.05 ± 1.05	0.75 (0.52–1.14)	0.674
*Prevotella*	0.28 ± 0.54	0.00 (0.00–0.43)	0.32 ± 1.27	0.01 (0.00–0.01)	0.795
*Atopobium*	0.03 ± 0.06	0.01 (0.00–0.02)	0.30 ± 1.14	0.02 (0.00–0.13)	0.091

^#^ Mann-Whitney U test. * *p* < 0.05 indicated the significant difference. IQR: interquartile range.

**Table 3:** Dietary pattern in 48 subjects.

Item		Eczema		
	All (*n* = 48)	No (*n* = 24)	Yes (*n* = 24)	*p*-Value
How many servings of fruit did you have every day on average during pregnancy (including all kinds of fresh, frozen, canned or dried fruits but excluding fruit juice and fruit desserts like mango pancake, apple pie, etc.)?				
None, or less than 1 serving per day	7 (14.6%)	4 (16.7%)	3 (12.5%)	0.385 ^ψ^
1 serving per day	16 (33.3%)	10 (41.7%)	6 (25.0%)	
1 serving per day	25 (52.1%)	10 (41.7%)	15 (62.5%)	
How many glasses of fruit juice did you drink every day on average during pregnancy (excluding sugar-added fruit juice)?				
None, or less than 1 glass per day	47 (97.9%)	24 (100.0%)	23 (95.8%)	0.999 ^ψ^
1 glass per day	0 (.0%)	0 (0.0%)	0 (0.0%)	
2 glasses (or more) per day	1 (2.1%)	0 (0.0%)	1 (4.2%)	
How many servings of vegetables did you have every day on average during pregnancy (including fresh, frozen or canned vegetables but excluding vegetable juice)?				
None, or less than 1 serving per day	3 (6.3%)	1 (4.2%)	2 (8.3%)	0.365 ^ψ^
1 serving per day	18 (37.5%)	12 (50.0%)	6 (25.0%)	
2 servings per day	18 (37.5%)	8 (33.3%)	10 (41.7%)	
3 servings (or more) per day	9 (18.8%)	3 (12.5%)	6 (25.0%)	
How many tael(s) * of meat, fish and eggs did you eat every day during pregnancy?				
None, or less than 1 tael	1 (2.1%)	0 (.0%)	1 (4.2%)	0.999 ^ψ^
1 to 2 taels per day	6 (12.5%)	3 (12.5%)	3 (12.5%)	
3 to 4 taels per day	24 (50.0%)	12 (50.0%)	12 (50.0%)	
5 taels (or more) per day	17 (35.4%)	9 (37.5%)	8 (33.3%)	
How many servings of bean products did you have every day on average during pregnancy?				
None, or less than half serving per day	28 (58.3%)	14 (58.3%)	14 (58.3%)	0.999 ^ψ^
Half a serving per day	5 (10.4%)	3 (12.5%)	2 (8.3%)	
1 serving per day	11 (22.9%)	5 (20.8%)	6 (25.0%)	
2 servings (or more) per day	4 (8.3%)	2 (8.3%)	2 (8.3%)	
How many dairy products did you have every day during pregnancy?				
None, or less than 1 glass per day	17 (35.4%)	13 (54.2%)	4 (16.7%)	0.007 ^ψ^
1 glass per day	26 (54.2%)	8 (33.3%)	18 (75.0%)	
2 glasses (or more) per day	5 (10.4%)	3 (12.5%)	2 (8.3%)	
How often did you eat stir-/deep-fried food or food with a large amount of oil during pregnancy (e.g., French fries, fried rice/noodles, sweet and sour pork, tempura, etc.)?				
None, or less than once a day	41 (85.4%)	21 (87.5%)	20 (83.3%)	0.999 ^ψ^
Once a day	7 (14.6%)	3 (12.5%)	4 (16.7%)	
How often did you have desserts or sugary foods and drinks during pregnancy (e.g., soft drinks, ice cream, chocolate, sweets, cake, pineapple buns, sugar-added coffee or tea, etc.)?				
None, or less than once a day	29 (60.4%)	14 (58.3%)	15 (62.5%)	0.901 ^ψ^
Once a day	14 (29.2%)	8 (33.3%)	6 (25.0%)	
Twice a day	4 (8.3%)	2 (8.3%)	2 (8.3%)	
3 times (or more) a day	1 (2.1%)	0 (0.0%)	1 (4.2%)	
How many serving(s) of grains did you eat every day on average during pregnancy (including pasta; noodles; oatmeal; rice; bread; biscuits or starchy vegetables such as potatoes, taro, etc.)?				
None, or less than 1 serving per day	1 (2.1%)	0 (0.0%)	1 (4.2%)	0.057 ^ψ^
1 serving per day	9 (18.8%)	2 (8.3%)	7 (29.2%)	
2 servings per day	22 (45.8%)	15 (62.5%)	7 (29.2%)	
3 servings (or more) per day	16 (33.3%)	7 (29.2%)	9 (37.5%)	
How many serving(s) of whole grains did you eat every day on average during pregnancy (including oatmeal, rice (red or brown), whole meal bread, high-fiber biscuits, etc.)?				
None, or less than 1 serving per day	38 (79.2%)	20 (83.3%)	18 (75.0%)	0.477 ^#^
1 serving per day	10 (20.8%)	4 (16.7%)	6 (25.0%)	

Data are presented as frequency (%). ^#^ Continuous and categorical variables were compared between the two groups using independent *t*-test and Pearson chi-square test, respectively. Those marked with ^ψ^ were compared using Fisher’s exact test. * Tael is a common weight-measuring unit in Hong Kong. 1 tael is approximately equal to 37.8 g.

**Table 4:** Dietary practices in 48 subjects.

Item		Eczema		
	All (*n* = 48)	No (*n* = 24)	Yes (*n* = 24)	*p*-Value
Which of the following describe most accurately your daily dietary practices during pregnancy (excluding snacks at tea time or at night)?				
3 regular meals daily	31 (64.6%)	15 (62.5%)	16 (66.7%)	0.999 ^ψ^
3 meals daily but taken irregularly	13 (27.1%)	6 (25.0%)	7 (29.2%)	
1 to 2 meals daily at regular hours	1 (2.1%)	1 (4.2%)	0 (.0%)	
Irregular meals	3 (6.3%)	2 (8.3%)	1 (4.2%)	
How often did you have snacks apart from regular meals during pregnancy (including at tea time and at night)?				
None, or less than once a day	22 (45.8%)	11 (45.8%)	11 (45.8%)	0.752 ^ψ^
Once a day	22 (45.8%)	10 (41.7%)	12 (50.0%)	
Twice (or more) a day	4 (8.3%)	3 (12.5%)	1 (4.2%)	
Which of the following describes most accurately your breakfast habits during pregnancy?				
No breakfast at all	1 (2.1%)	0 (.0%)	1 (4.2%)	0.999 ^ψ^
No breakfast most of the time	2 (4.2%)	1(4.2%)	1 (4.2%)	
Breakfast most of the time	45 (93.8%)	23 (95.8%)	22 (91.7%)	
How many meals (including breakfast, lunch, dinner and tea time and night snacks) did you or your family members make every day on average during your pregnancy?				
Less than 1 meal per day	4 (8.3%)	3 (12.5%)	1 (4.2%)	0.752 ^ψ^
1 meal per day	17 (35.4%)	9 (37.5%)	8 (33.3%)	
2 meals per day	17 (35.4%)	8 (33.3%)	9 (37.5%)	
3 meals (or more) per day	10 (20.8%)	4 (16.7%)	6 (25.0%)	
Did you take any supplements during pregnancy?				
No	5 (10.4%)	3 (12.5%)	2 (8.3%)	0.037 ^ψ^
Yes, <3 months	4 (8.3%)	4 (16.7%)	0 (.0%)	
Yes, 3–6 months	4 (8.3%)	1 (4.2%)	3 (12.5%)	
Yes, 6–9 months	22 (45.8%)	13 (54.2%)	9 (37.5%)	
Yes, 9–12 months	13 (27.1%)	3 (12.5%)	10 (41.7%)	

Data are presented as frequency (%). ^ψ^ Variables were compared between the two groups using Fisher’s exact test.

**Table 5:** Allergy data of 48 subjects.

Item		Eczema		
	All (*n* = 48)	No (*n* = 24)	Yes (*n* = 24)	*p*-Value
Family history of eczema				
No	33 (68.8%)	18 (75.0%)	15 (62.5%)	0.350 ^#^
Yes	15 (31.3%)	6 (25.0%)	9 (37.5%)	
Family history of asthma				
No	43 (89.6%)	21 (87.5%)	22 (91.7%)	0.999 ^ψ^
Yes	5 (10.4%)	3 (12.5%)	2 (8.3%)	
Family history of pollen allergy				
No	46 (95.8%)	23 (95.8%)	23 (95.8%)	0.999 ^ψ^
Yes	2 (4.2%)	1 (4.2%)	1 (4.2%)	
Family history of food allergy				
No	40 (83.3%)	21 (87.5%)	19 (79.2%)	0.701 ^ψ^
Yes	8 (16.7%)	3 (12.5%)	5 (20.8%)	
Have you breastfed (for any length of time), or are you breastfeeding your child now?				
No	7 (14.6%)	5 (20.8%)	2 (8.3%)	0.416 ^ψ^
Yes	41 (85.4%)	19 (79.2%)	22 (91.7%)	
Was your child taking formula, as either the main or complementary food (for any time)?				
No	40 (83.3%)	19 (79.2%)	21 (87.5%)	0.701 ^ψ^
Yes	8 (16.7%)	5 (20.8%)	3 (12.5%)	
Were you smoking during pregnancy?				
No	46 (95.8%)	22 (91.7%)	24 (100.0%)	0.489 ^ψ^
Yes	2 (4.2%)	2 (8.3%)	0 (0.0%)	
Does anybody currently smoke inside your child’s home?				
No	35 (72.9%)	19 (79.2%)	16 (66.7%)	0.330 ^#^
Yes	13 (27.1%)	5 (20.8%)	8 (33.3%)	
Did you receive any antibiotic treatment during pregnancy?				
No	37 (77.1%)	17 (70.8%)	20 (83.3%)	0.494 ^ψ^
Yes	10 (20.8%)	6 (25.0%)	4 (16.7%)	
Do not know	1 (2.1%)	1 (4.2%)	0 (.0%)	
Did you have any pet at home during pregnancy?				
No	33 (68.8%)	20 (83.3%)	13 (54.2%)	0.029 ^#^
Yes	15 (31.3%)	4 (16.7%)	11 (45.8%)	
Did you have any furry pet at home during pregnancy?				
No	35 (72.9%)	20 (83.3%)	15 (62.5%)	0.104 ^#^
Yes	13 (27.1%)	4 (16.7%)	9 (37.5%)	
Do you have any pet at home now?				
No	38 (79.2%)	21 (87.5%)	17 (70.8%)	0.155 ^#^
Yes	10 (20.8%)	3 (12.5%)	7 (29.2%)	
Do you have any furry pet at home now?				
No	39 (81.3%)	21 (87.5%)	18 (75.0%)	0.461 ^ψ^
Yes	9 (18.8%)	3 (12.5%)	6 (25.0%)	
Did a doctor ever say you had an infection during pregnancy?				
No	29 (60.4%)	14 (58.3%)	15 (62.5%)	0.768 ^#^
Yes	19 (39.6%)	10 (41.7%)	9 (37.5%)	
What is your rating of the air quality in the area where you live?				
Good	26 (54.2%)	13 (54.2%)	13 (54.2%)	0.999 ^ψ^
Moderate	20 (41.7%)	10 (41.7%)	10 (41.7%)	
Bad	2 (4.2%)	1 (4.2%)	1 (4.2%)	

Data are presented as frequency (%). ^#^ Continuous and categorical variables were compared between the two groups using independent *t*-test and Pearson chi-square test, respectively. Those marked with ^ψ^ were compared using Fisher’s exact test.

**Table 6:** Factors associated with the occurrence of eczema.

Item	Eczema					
	No (*n* = 24)	Yes (*n* = 24)	OR_U_	*p*	OR_A_ (95% CI)	*p*
Father’s education						
Secondary	15 (71.4%)	6 (28.6%)	1		1	
Tertiary	9 (33.3%)	18 (66.7%)	5.00	0.011	9.93 (1.83–53.71)	0.008
Dairy products consumed daily during pregnancy						
<1 glass	13 (76.5%)	4 (23.5%)	1		NS	
≥1 glass	11 (35.5%)	20 (64.5%)	5.91	0.009		
Cereal food consumed daily during pregnancy						
≤1 proportion	2 (20.0%)	8 (80.0%)	1		1	
≥2 proportions	22 (57.9%)	16 (42.1%)	0.18	0.046	0.10 (0.01–0.82)	0.032
Having taken nutritional supplements for more than 9 months during pregnancy						
No	21 (60.0%)	14 (40.0%)	1		1	
Yes	3 (23.1%)	10 (76.9%)	4.99	0.030	10.75 (1.57–73.46)	0.015
Having pets at home during pregnancy						
No	20 (60.6%)	13 (39.4%)	1		NS	
Yes	4 (26.7%)	11 (73.3%)	4.23	0.035		

ref: Reference group of the categorical variable. OR_U_: univariate odds ratio. OR_A_: odds ratio adjusted for other significant factors obtained from the backwards stepwise logistic regression analysis using variables with *p*-values < 0.1 in univariate analysis as candidate variables. NS: not statistically significant in the multivariable analysis.
